# Morphological and Optical Characteristics of Chitosan_(1−*x*)_:Cu^o^*_x_* (4 ≤ *x* ≤ 12) Based Polymer Nano-Composites: Optical Dielectric Loss as an Alternative Method for Tauc’s Model

**DOI:** 10.3390/nano7120444

**Published:** 2017-12-13

**Authors:** Shujahadeen B. Aziz

**Affiliations:** 1 Advanced Polymeric Materials Research Laboratory, Department of Physics, College of Science, University of Sulaimani, Sulaimani 46001, Kurdistan Regional Government, Iraq; shujahadeenaziz@gmail.com or shujahadeen.aziz@univsul.edu.iq; 2 Komar Research Center (KRC), Komar University of Science and Technology, Sulaimani 46001, Kurdistan Regional Government, Iraq

**Keywords:** biopolymer, Cu nanoparticles, SEM and EDAX analysis, TEM analysis, optical properties

## Abstract

In this work, copper (Cu) nanoparticles with observable surface plasmonic resonance (SPR) peaks were synthesized by an in-situ method. Chitosan host polymer was used as a reduction medium and a capping agent for the Cu nanoparticles. The surface morphology of the samples was investigated through the use of scanning electron micrograph (SEM) technique. Copper nanoparticles appeared as chains and white specks in the SEM images. The strong peaks due to the Cu element observed in the spectrum of energy dispersive analysis of X-rays. For the nanocomposite samples, obvious peaks due to the SPR phenomena were obtained in the Ultraviolet-visible (UV-vis) spectra. The effect of Cu nanoparticles on the host band gap was understood from absorption edges shifting of absorption edges to lower photon energy. The optical dielectric loss parameter obtained from the measurable quantities was used as an alternative method to study the band structure of the samples. Quantum mechanical models drawbacks, in the study of band gap, were explained based on the optical dielectric loss. A clear dispersion region was able to be observed in refractive indices spectra of the composite samples. A linear relationship with a regression value of 0.99 was achieved between the refractive index and volume fractions of CuI content. Cu nanoparticles with various sizes and homogenous dispersions were also determined from transmission electron microscope (TEM) images.

## 1. Introduction

A recent study revealed that nano-scale materials exhibit unique electronic and optical properties, which are unlike those in their bulk state [1]. Metallic nanoparticles have attracted attention of many researchers, because of the local field enhancement at nano interfaces, which is beneficial for a number of applications, from sensors to nonlinear optics [2]. Plasmonic metal nanoparticles are described by their strong interactions with UV-visible radiation through the localized surface plasmon resonance (LSPR) excitation [3]. Copper (Cu) is the most commonly used metal in electrical/electronic applications, because of its high conductivity and low cost. The development of nano-devices that combine electronic, photonic, chemical and/or biological features is crucial for future electronic and sensing devices [4]. The importance of copper nanoparticles (CuNPs) arises from the advantageous properties of this metal, such as its good thermal and electrical conductivities at a cost much less than that of silver. This leads to potential applications in cooling fluids for electronic systems and conductive inks [5]. Copper “incorporated into” or “supported on” solid matrices is broadly utilized in the catalysts and nanocomposites preparations with unusual optical, electrical and magnetic properties [6]. The photosensitivity of noble metal nanostructures makes them promising platforms for highly sensitive optical nanosensors, photonic components and surface-enhanced spectroscopies [4]. Nanoparticles have been synthesized through several methods, such as the polyol, reverse micelles, electron beam irradiation, micro-emulsion and wire explosion techniques and in-situ chemical synthesis. Among all the procedures, a compound that has the ability to form a complex with metal ions, such as soluble polymers, is an important method for CuNPs synthesis, since it prevents the nanoparticles aggregation [7,8]. Previous work indicated that physical and chemical methods have been used to synthesize polymer nanocomposites, depending on the nanoparticle-polymer interactions [9]. The usage of synthetic polymers in general and polymer composites in particular appears to be ever increasing. However, despite the fact that synthetic polymer and their composite are cost effective; they are non-biodegradable materials and produced from petroleum sources. On the other hand, biodegradable polymers produced from renewable sources are cheap and easy to treat without any hazardous chemicals [10]. Recently, the use of natural bio-polymers as stabilizers for the synthesis of CuNPs has been gaining momentum because of their availability, biocompatibility and low toxicity [11]. Chitosan (CS) has attracted significant attention as functional, nontoxic and biodegradable natural biopolymer for many applications [12]. Chitosan is a cationic polysaccharide and obtained by alkaline *N*-acetylation of chitin, which is the second-most abundant natural polymer after cellulose [13]. The amine (NH_2_) and hydroxyl (OH) functional groups on the CS backbone structure explain its ability to form complexes with inorganic salts [12]. Previous studies confirmed that the existences of lone pair electrons on chitosan functional groups are found to be responsible for complexation as well as reduction of silver ions to silver nanoparticles [14,15,16,17,18]. However, such problem can be overcome by the use of in-situ technique [19]. Thus, the direct use of chitosan can solve the problems of aggregation. Earlier study revealed that hybrid (organic-inorganic) materials represents an intrinsic interdisciplinary field of research and development because it includes a variety of communities such as organometallics, colloids, soft matter, polymers, nanocomposites, biomaterials and biochemistry [20]. This is related to the fact that organic-inorganic materials lie at the interface of the organic and inorganic areas. These materials present outstanding chance not only to combine the fundamental properties from both worlds but to create entirely new compositions with exclusive properties [21]. The optical properties of hybrid materials are currently of considerable interest, due to their wide applications in sensors, single-molecule detection, optical data storage and light-emitting diodes (LED) [22,23]. Moreover, recent study indicated that hybrid materials are crucial in the development of numerous types of organic transistors, organic light-emitting diodes and organic solar cells [24].The intensive and extensive survey of literature reveals that band gap study of polymer composites are not studied in detail. The objective of this work was to study the optical properties of synthesized Cu nanoparticles via the in-situ method inside the chitosan host polymer at room temperature. The results shown in this paper reveals that a Cu nanoparticle significantly reduces the optical band gap of chitosan host polymer. The Cu-chitosan polymer composites were characterized using scanning electron micrograph (SEM), energy dispersive analysis of X-rays (EDAX), transmission electron microscopy (TEM) and UV-visible techniques. The effect of CuNPs on the optical properties of the chitosan host polymer was clarified.

## 2. Experimental Details

### 2.1. Materials and Sample Preparation 

Chitosan (≥75% deacetylated, molecular weight *M*_w_ = 1.1 × 10^5^) and copper iodide (CuI) were supplied by Sigma Aldrich (Sigma Aldrich, Warrington, PA, USA). Acetic acid (1%) was prepared using glacial acetic acid solution and used as a solvent to prepare the nanocomposite solid polymer electrolytes (SPEs). The standard solution cast technique was used to prepare the SPE films. Here, 1 g of chitosan was dissolved in 100 mL of 1% acetic acid solution. The mixture was stirred continuously with a magnetic stirrer for several hours at room temperature until the chitosan powder was completely dissolved in the 1% acetic acid solution. Different amounts (4 to 12 wt. %) of CuI were dissolved in 20 mL of acetonitrile (CH_3_CN) solvent. To prepare nanocomposite samples, the CuI solution was added to the chitosan solutions separately and the mixture was stirred continuously for several hours. The solutions were then cast into different clean and dry Petri dishes and allowed to evaporate at room temperature until solvent-free films were obtained. The films were kept in desiccators with blue silica gel desiccant for further drying. The greenish colors of the chitosan nanocomposite films are evidence of the formation of Cu nanoparticles. The thickness of the films ranged from 121–123 μm was controlled by casting the same amount of CS. The chitosan nanocomposite (CSN) samples were coded as CSN0, CSN1, CSN2 and CSN3 for CS incorporated with 0 wt. %, 4 wt. %, 8 wt. % and 12 wt. % CuI, respectively.

### 2.2. Characterization Techniques

The UV-visible spectra of the chitosan-silver triflate membrane films and their nanocomposites were recorded, using a Jasco V-570 UV-Vis-NIR spectrophotometer (Jasco SLM-468, Tokyo, Japan) in the absorbance mode. Scanning electron microscopy (SEM) was taken to study the morphological appearance of the samples, using the FEI Quanta 200 Field Emission Scanning Electron Microscopy (FESEM) (FEI Company, Hillsboro, OR, USA). The microscope was fitted with an Oxford instruments INCA Energy 200 energy-dispersive X-ray microanalysis (EDXA) system (Abingdon, UK) [Detector: Si (Li) crystal] to detect the overall chemical composition of solid chitosan nanocomposites. Transmission electron microscope (TEM) image was also obtained for the Cu nanoparticles (CSN3 sample), using a LEO LIBRA instrument (Carl Zeiss, Oberkochen, Germany)with accelerating voltage of 120 kV. For TEM measurement, a drop of chitosan:CuI solution containing Cu nanoparticles was placed on a carbon-coated copper grid. The excess solution was then removed by filter paper and the grid was left at room temperature to dry prior being imaged.

## 3. Results and Discussion

### 3.1. Morphological Studies

**Figure 1:**
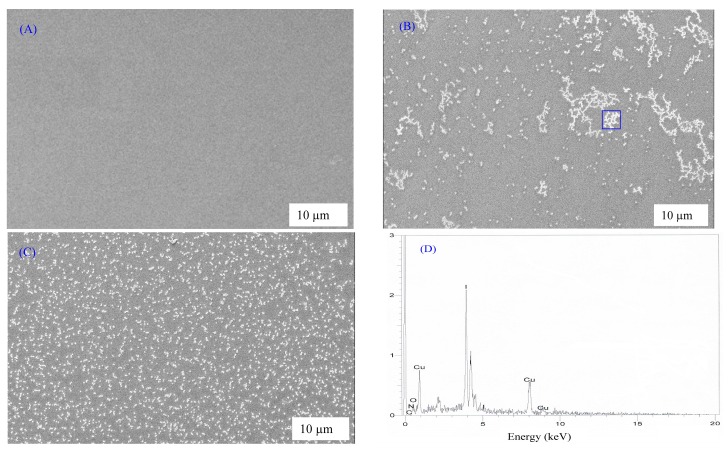
Scanning electron microscopy (SEM) image for (**A**) CSN1; (**B**) CSN2; (**C**) CSN3 and (**D**) EDAX for white specks inside the blue box. The white spots appearing on the film surface are attributable to Cu metallic particles.

Figure 1 shows the SEM images and EDAX results for the CS nanocomposite samples. It is clear from the figures that, at high CuI concentration, white chains and spots with different sizes can be observed on the surface of the CS nanocomposite samples. Earlier studies have confirmed that the use of SEM and EDAX techniques are sufficient to detect the formation of plasmonic metal nanoparticles in polymer composites [13,14,15,17,18]. The EDAX taken for the CSN2 sample focusing on the aggregated white spots is illustrated in Figure 1D, which shows the existence of significant amounts of metallic Cu particles. The electron image was taken at 100× magnifications and shows very small white specs for CSN1 sample, while many white aggregates can be seen for images of CSN2 and CSN3 samples. The SEM-EDAX results presented here confirm the successful formation of Cu nanoparticles via the in-situ method inside the chitosan host polymer. Such results can be further examined by the use of UV-visible and TEM techniques. The results of the present work suggest that chitosan can be used as a novel polar polymer for synthesis of plasmonic metallic nanoparticles. The distinguishable intense peaks of Cu nanoparticles appeared in the EDAX spectrum at approximately 1 and 8 keV confirms the formation of Cu nanoparticles. Similar peaks for Cu nanoparticles in EDAX spectra have been observed by other researchers [25,26,27]. From this discussion, it is understood that morphological (SEM and EDAX) study is significant for nanoparticle characterization.

### 3.2. Optical Properties

#### 3.2.1. Absorption and Absorption Edge Study

**Figure 2:**
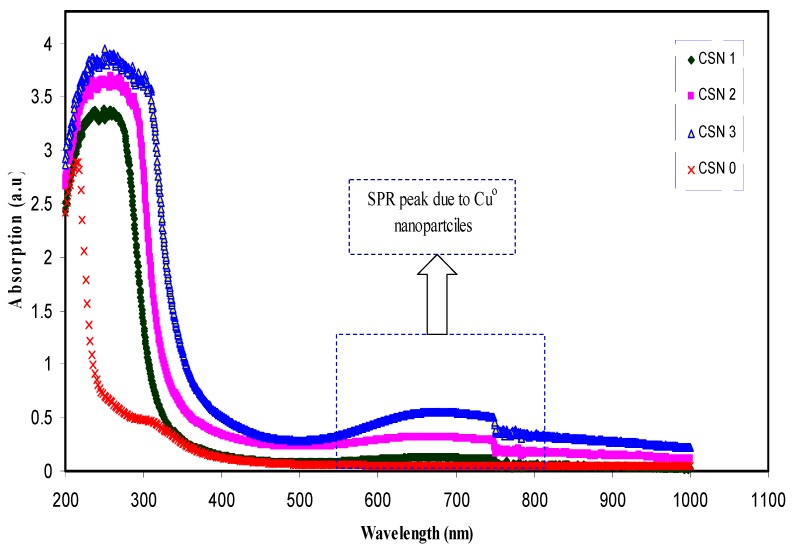
Absorption spectra of pure CS and CS:CuI solid films. The surface plasmonic resonance (SPR) peak appearing at approximately 667 nm for CS:CuI samples is related to the existence of Cu metallic nanoparticles.

Figure 2 shows the absorption spectra of pure CS and CS nanocomposite samples. It can be seen that the pure CS does not indicate any absorption peak at 644 nm, whereas the samples incorporated with CuI exhibit distinguishable peaks at wavelength ranges from 550 to 750 nm. These peaks can only be attributable to the LSPR excitation that occurs due to the nanoscale-size metal particles [12,28]. An earlier study established that the LSPR band occurring near 620–640 nm is related to the formation of copper and copper oxide nanoparticles [29]. On the other hand, in our previous works, we have observed distinguishable and enhanced LSPR peaks for CuO- and CuS-based nanoparticles [29,30,31]. Therefore, the SPR peaks presented in this work can be attributed to the Cu nanoparticles alone. It is interesting to note that the results of absorption spectra achieved in this work are close to those reported by other researchers [2,8]. The shift of absorption spectra to the visible ranges for the nanocomposite samples reveals the role of Cu nanoparticles on optical properties of CS host polymer. More insights about the change in the band structure of chitosan can be grasped from the band gap study. Earlier researchers used ion implantation to fabricate gold (Au)-polyimide hybrid. They achieved SPR peaks with low intensity and they do not study the effect of Au nanoparticles on optical properties of polyimide [32]. Significant shifting of absorption spectra to visible region reveals that Cu nanoparticles forms charge transfer complexes through the CS host polymer. The results of the current work show that polymer composites incorporated with Cu nanoparticles possess great changes in their optical properties compared to hybrid materials containing silver nanoparticles [33,34,35]. It is obvious that around 750 nm an artifact or a step rise occurs. The appearance of artifact around 750 nm may be related to the change of the range of measurement from visible to infrared IR region [12].

**Figure 3:**
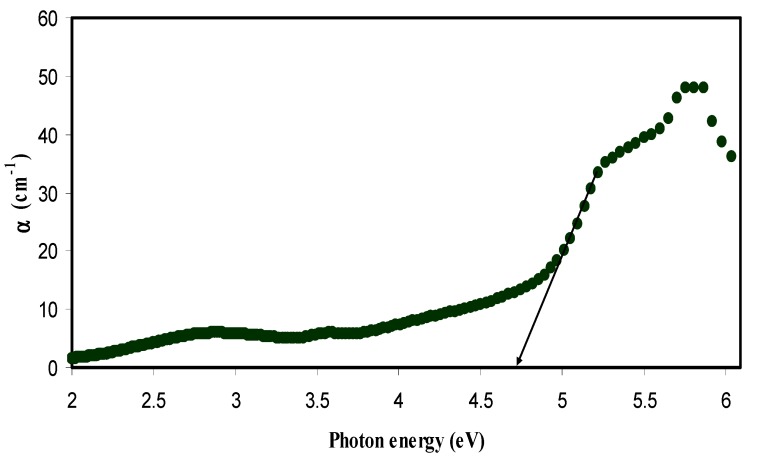
Variation of absorption coefficient (α) versus photon energy (hυ) for pure CS sample.

**Figure 4:**
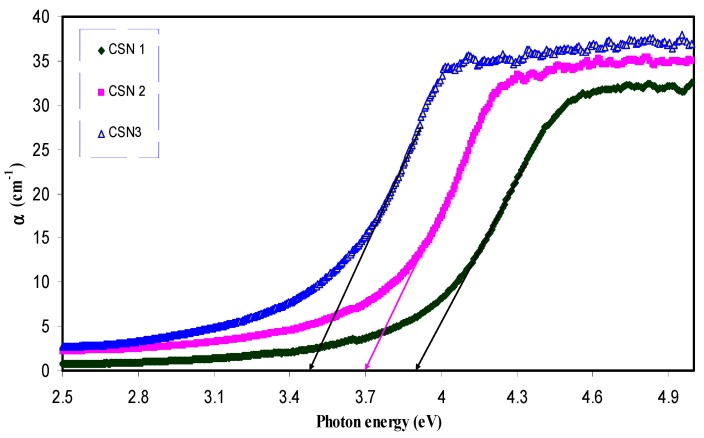
Variation of absorption coefficient (α) versus photon energy (hυ) for doped samples.

The absorption of light by an optical medium is quantified by its absorption coefficient (*α*) [36]. The absorption coefficient is calculated by,

(1) $$\alpha_{\left(\lambda \right)}=(2.303)\times [\frac{A}{d}]$$

where *d* is the thickness of the sample and *A* is the absorption data. Figure 3 and Figure 4 illustrate the absorption coefficient as a function of photon energy for pure CS and CS nanocomposite samples, respectively. Absorption coefficient is defined as the fraction of the power absorbed in a unit length of the medium [36]. It can be clearly seen that the absorption edge of the CS shifts toward the lower photon energy for the samples containing Cu nanoparticles. Essential information about the band structure and the energy band gap in the crystalline and non-crystalline materials can be obtained from the optical absorption spectra determination [37]. The shift towards the lower photon energy reveals the reduction in the optical band gap of the CS nanocomposite samples. Absorption edge values were estimated from the intersection of the extrapolation of the linear relationship. The obtained absorption edge values are presented in Table 1. It is clear that the absorption edge decreases from 4.7 eV for pure CS to 3.51 eV for CS incorporated with 12 wt. % CuI. Such significant changes in absorption edge values are evidence of the occurrence of changes in the band structure of the CS nanocomposite samples.

#### 3.2.2. Refractive Index and Optical Dielectric Constant Study

**Figure 5:**
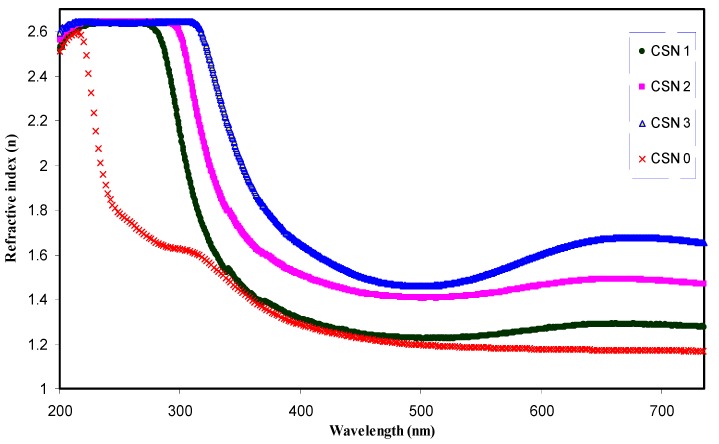
Refractive index (n) as a function of wavelength for pure CS and doped CS samples.

**Figure 6:**
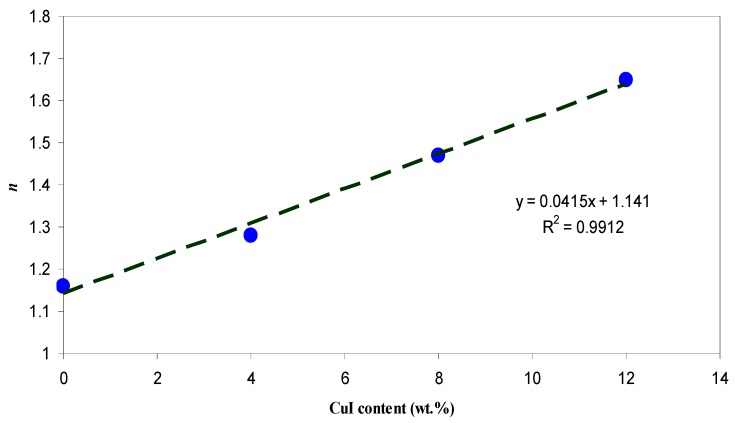
Refractive index (n) as a function of volume fraction of CuI.

**Figure 7:**
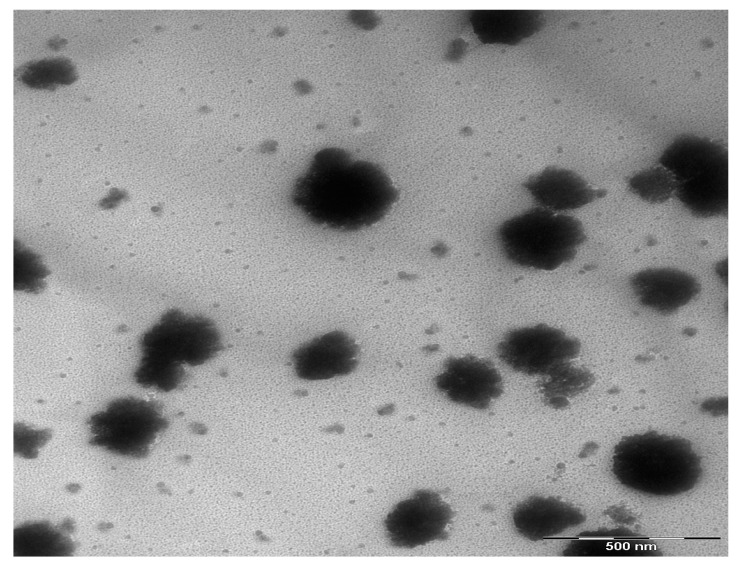
TEM image for CSN3 samples.

Changes in the refractive index of composite films are crucial for controlling optical properties of materials. An earlier study revealed that in refractive index analysis, the real part (*ε*_1_) and imaginary part (*ε*_2_) of the optical dielectric constant are important for designing new materials. Refractive index is an important optical parameter for designing prisms, optical windows and optical fibers [38]. From the reflection coefficient *R* and the optical extinction data, the refractive indexes (*n*) can be estimated, using the Fresnel formulae as follows [28]:

(2) $$n=\left(\frac{1+R}{1-R}\right)+{\left[\frac{4R}{{\left(1-R\right)}^{2}}-K^{2}\right]}^{1/2}$$

where *R* is reflection coefficient and *K* = *αλ*/4π is the extinction coefficient. Figure 5 illustrates the refractive index of pure CS and CS nanocomposite samples. A significant change in refractive index has been occurred for the doped samples. It is obvious that the refractive index increased from 1.16 for pure CS to 1.68 for CS incorporated with 12 wt. % CuI. The synthesis of Cu nanoparticles through the host polymer makes the polymer matrix denser in nature and thus exhibits high refractive index according to the well-known Lorentz–Lorenz formula [31,39]. Materials with high refractive index play an important role in many top end advanced optical and optoelectronic equipment, including waveguides, antireflective coatings and light-emitting diodes [40]. It is evident from Figure 5 that the dispersion curve becomes steeper for samples containing a higher concentration of Cu nanoparticles. The extension of plateau region of Figure 5 at high wavelengths to the *Y*-axis was used to estimate the index of refraction. It is clear that the refractive index increases linearly with Cu concentration, as shown in Figure 6. The linear nature of the refractive index with filler dopant has been reported experimentally and theoretically [28,30,36,41,42]. Earlier studies established that the linear dependence of the refractive index indicates the homogeneous dispersion of fillers within a polymer matrix [28,30,36,42]. This is further illustrated in the transmission electron microscopy (TEM) image Figure 7, which taken for the CSN3 sample. Here, the homogeneous dispersion of Cu nanoparticles with various sizes can be seen. The linear behavior of the refractive index and TEM result indicates that the in-situ method is an excellent technique for the preparation of polymer nanocomposites with homogeneous dispersion of fillers in the matrices.

**Figure 8:**
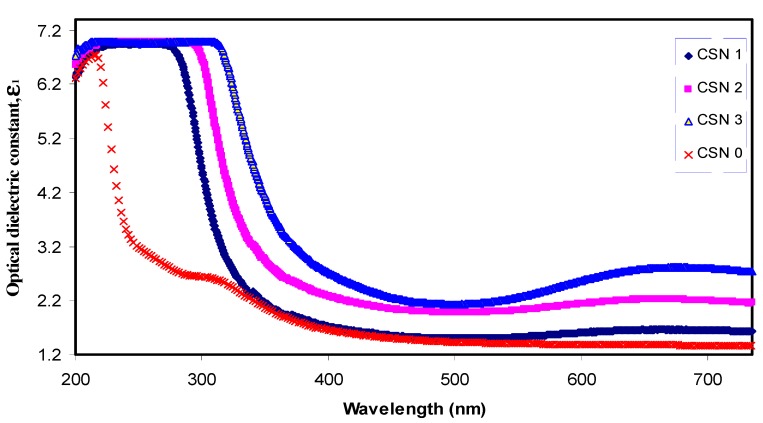
Optical dielectric constant spectra versus wavelength for pure CS and doped CS samples.

Figure 8 shows the optical dielectric constant (*ε*_1_) versus wavelength. From the graph, an obvious increase in dielectric constant upon the incorporation of CuI content can be seen. Such an increase is related to the increment of the density of states since *ε*_1_ can be directly associated with the density of states inside the forbidden gap of the solid polymer films [43]. Previous studies established that *ε*_1_ is related to the electronic part and depends strongly on the optical bandgap [28,44]. This can be better understood from the well-known Penn model [45], as given by:

(3) $$\varepsilon_{1}(0)\approx 1+{\left(\hbar \omega_{p}/E_{g}\right)}^{2}$$

It is evident from Equation (3) that a smaller energy gap (*E_g_*) yields a larger *ε*_1_ value. The *ε*_1_ and *n* values are presented in Table 1. Here, it is clear that the reduction in optical band gap is associated with the increase in refractive index. The decrease in the optical band gap (see Table 1) can be related to an increase in optical dielectric constant. An increase in optical dielectric constant means the introduction of more charge carriers to the host material and thus an increase in the density of states [46]. The results obtained in this work reveal the validity of the Penn model, in which the increment of the density of states within the band gap causes the energy band gap to be increased. It can be observed that the values of *ε*_1_ (see Table 1) increase with the square of refractive index, which exactly meets the relation of *ε*_1_ *= n*^2^. 

#### 3.2.3. Band Gap Study

In the present work, based on experimental and theoretical approaches, two methods were performed for the band-gap study. Tauc’s method has been developed from the theory of optical absorption. It is a familiar method for the study of band-gap, which relates the absorption coefficient to the photon energy. On the other hand, theoretical physicists have developed various models based on quantum approaches for band-gap study. Theoretically, all intrinsic effects corresponding to light-matter interaction processes are contained in the optical dielectric function [47]. It is recognized from the previous studies that the main peak of optical dielectric loss (*ε*_2_) is directly related to the electron transitions from valence band to conduction band [28,46,48]. Therefore, the optical dielectric loss parameter versus photon energy can be considered for band gap study. 

It was reported that the band edges, in the amorphous materials, have contributions from the different orbital types of the metallic complex and ligand. Therefore, it is difficult to predict whether the band will be a direct or an indirect type [15,49]. In this regard, it should be pointed out that the Tauc’s equation alone is not enough to specify the type of transition, since four figures is needed to be plotted depending on the values that the exponent takes [15]. From a quantum mechanics viewpoint, the existence of strong correlation between optical dielectric loss (*ε*_2_) and band structure of the materials is a subject of various theoretical studies [46,50,51,52]. The use of *ε*_2_ to estimate the optical band gap, is related to the fact that *ε*_2_ is a function of the electronic band-structure, density of filled and empty states and magnitude of optical transition probability between the filled and empty states [53,54], as follows:

(4) $$\varepsilon_{2}(\omega )=\frac{4\pi^{2}}{\Omega \omega^{2}}{\sum_{i\in VB.j\in CB}\sum_{K}W_{K}\left|p_{ij}^{a}\right|}^{2}\delta (\in_{kj}-\in_{ki}-\omega )$$

where Ω is the unit-cell volume and *ω* is photon frequency, VB and CB denote the valence and conduction bands, *W_k_* is the weight associated with a *k*-point, $p_{ij}^{a}$ is the transition probability and a denotes a particular direction. The delta function (δ) is used to ensure the conservation of energy in electronic transitions. The transition can occur only when the photon energy matches the energy difference between the valence and conduction state. Thus, from Equation (4) it is clear that optical dielectric loss is directly related to the material band structure and can be used to estimate the optical band gap.

The optical band gap has been studied by other researchers both experimentally and theoretically [50,54]. They observed that the experimental values for the band gap energy are found to be larger than the theoretical values. They have attributed this discrepancy to neglecting excitons and to poor description of strong Coulomb exchange interaction among electrons. The measurable optical dielectric loss has been used experimentally due to the shortcoming of the quantum models. In our previous works, the optical dielectric loss from the measured quantities, such as refractive index (*n*) and extinction (*K*) coefficient (*ε*_2_ = 2*nK*), has been successfully used to estimate the optical band gap. From the quantum mechanical viewpoint, Equation (4) was found to be a microscopic approach for the study of band-gap and required lengthy numerical methods. By contrast, it is found that using the experimental value of optical dielectric loss (*ε*_2_ *=* 2*nK*), which is a macroscopic approach, is easier and more accurate method to estimate the optical band gap [28,46]. 

**Figure 9:**
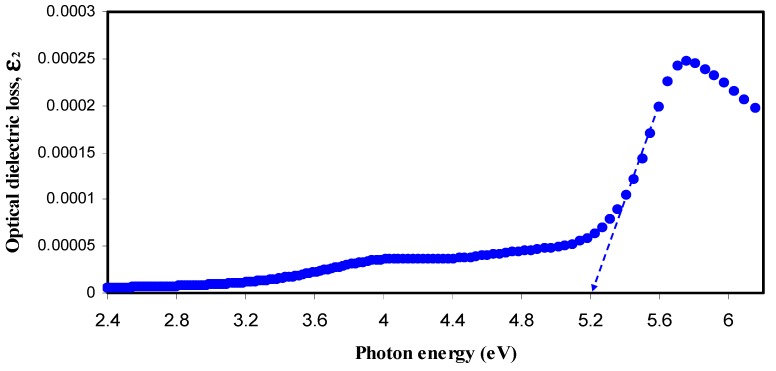
Optical dielectric loss versus photon energy (*hυ*) for pure CS sample.

**Figure 10:**
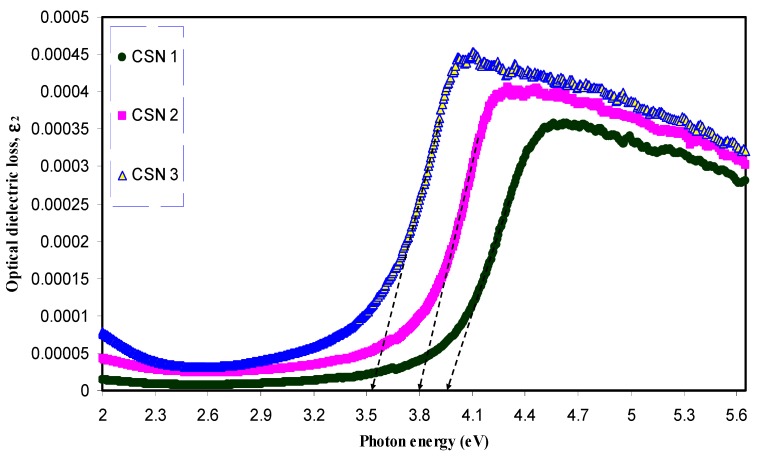
Optical dielectric loss versus photon energy (*hυ*) for doped CS samples.

Figure 9 and Figure 10 show the optical dielectric loss versus photon energy for pure- and doped-CS samples. It is well established that crystalline materials have sharp structures in the fundamental absorption region, whereas amorphous materials exhibit broad peaks [55]. Compared to the doped samples pure CS has exhibited a sharp peak. This is related to the existence of large amount of crystalline fraction in pure CS [56,57,58]. In our previous work, broad peaks in the plot of optical dielectric loss have been observed due to the amorphous nature of the composite samples [28,46]. The broad peaks depicted in Figure 10 may be ascribed to the amorphous structure of the samples. The estimated band gaps from the optical dielectric loss plots are presented in Table 2. To specify the type of electronic transition, Tauc’s method must be studied. 

The theory of optical absorption provides the relationship between the absorption coefficient (*α*) and photon energy (*hv*) for direct allowed transition [59]. The absorption coefficient for non-crystalline materials related to the incident photon energy can be determined as [28,60]:

(5) $$\alpha =\frac{\beta }{h\upsilon }{\left(h\upsilon -E_{g}\right)}^{\gamma }$$

where *β* is a constant and *E*_g_ is the optical energy bandgap. The exponent *γ* may have values of 1/2, 2, 3/2 and 3, which correspond to the allowed direct, allowed indirect, forbidden direct and forbidden indirect excitations, respectively [46]. Thus, from Tauc’s relation, four figures can be plotted to specify the type of transition. In this work, the plot corresponding to allowed direct excitation (*γ* = 1/2) is only presented due to that the plots corresponding to other values of *γ* exhibit optical band gaps that cannot be compared with those achieved from the optical dielectric loss plots (Figure 9 and Figure 10). 

**Figure 11:**
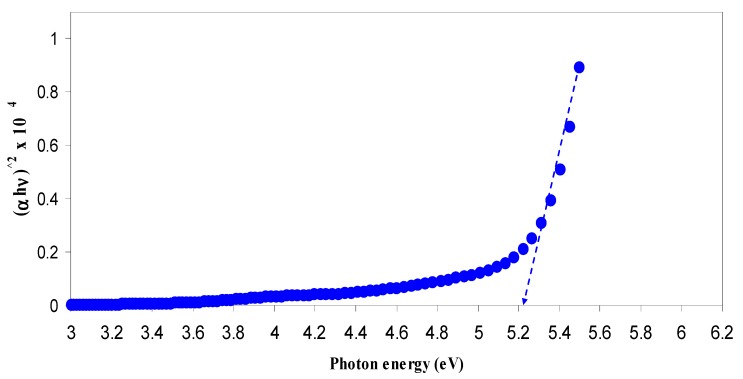
Plot of ${\left(\alpha h\upsilon \right)}^{2}$ versus photon energy (hυ) for pure CS sample.

**Figure 12:**
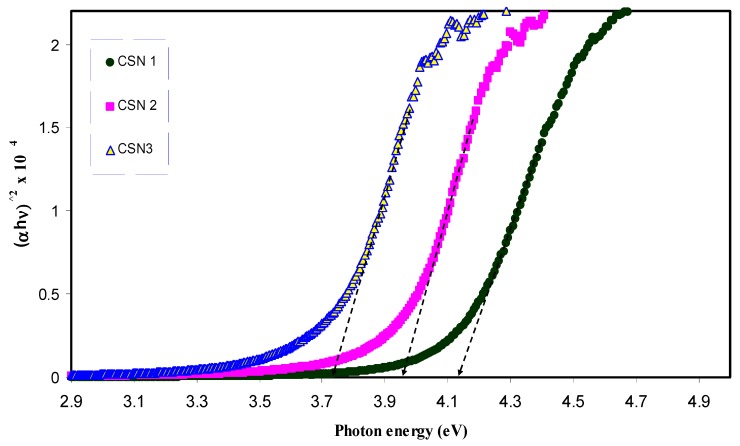
Plot of ${\left(\alpha h\upsilon \right)}^{2}$ versus photon energy (hυ) for doped CS samples.

Table 2 shows the band gap values estimated from the linear parts of Figure 11 and Figure 12. In the table, one can see that the band gap decreases with increasing CuI concentration. Previous studies concluded that an electronic interaction occurs between the nanoparticles and the host polar polymer and results in an increase in the absorption intensity [28,31,61]. This has been related to the fact that the embedded nanoparticles inside the host polymer may set up many localized charge carrier levels called trapping sites [31]. It is clear from Table 2 that the achieved optical band gaps estimated from Tauc’s model are close enough to those from the optical dielectric loss spectra. Thus, it is understood that optical dielectric loss is essential for band-gap study and Tauc’s method is an important way to determine the types of transition, by which the electron can cross the forbidden gap from the valence band to the conduction band. The band gap achieved from Tauc’s relation must be quite close to the expected values from optical dielectric loss. This is related to the main peak appearing in *ε*_2_ versus photon energy corresponds to the strong interband transitions [28].

## 4. Conclusions 

The results show that copper nanoparticles with observable SPR peaks can be synthesized by the in-situ method inside the chitosan host polymer. The white specks and chains of Cu nanoparticles were observed through SEM images. Strong peaks due to the Cu element appeared at approximately 1 and 8 keV in EDAX. Obvious peaks due to SPR phenomena were obtained in the UV-visible spectra of the nanocomposite samples. The effect of Cu nanoparticles on band gap of the host polymer could be expected from shifting of absorption edges to lower photon energy. The obtained optical dielectric loss parameter from measurable quantities was used to study the band structure of the samples. Tauc’s model was used to specify the electronic transition type. The drawbacks of the quantum models based on optical dielectric loss for band-gap study were explained. A clear dispersion region was observed in the spectra of the refractive indices. A linear relationship with a regression value of 0.99 between the refractive index and volume fractions of CuI content reveals the homogeneous distribution of Cu nanoparticles. The TEM image shows Cu nanoparticles with various sizes and homogeneous dispersions.

## Figures and Tables

**Table 1:** Refractive index and optical dielectric constant for all the samples.

Sample Designation	Estimated Refractive Index from Figure 5	Estimated Optical Dielectric Constant (*ε*_1_) from Figure 8	Estimated Refractive Index from *n =* (*ε*_1_)^1/2^
CSN0	1.16	1.36	1.16619
CSN1	1.28	1.63	1.276715
CSN2	1.47	2.17	1.473092
CSN3	1.65	2.76	1.661325

**Table 2:** Estimated energy band gap (*E*_g_) from optical dielectric loss and Tauc method for all samples.

Sample Designation	Estimated Bandgap (eV) from *ε*_2_ Plot	*E*_g_ (eV) from Tauc Method (*γ* = 1/2)
CSN0	5.2	5.24
CSN1	3.94	4.14
CSN2	3.8	3.95
CSN3	3.53	3.72
